# Using wearable cameras to categorise type and context of accelerometer-identified episodes of physical activity

**DOI:** 10.1186/1479-5868-10-22

**Published:** 2013-02-13

**Authors:** Aiden R Doherty, Paul Kelly, Jacqueline Kerr, Simon Marshall, Melody Oliver, Hannah Badland, Alexander Hamilton, Charlie Foster

**Affiliations:** 1 British Heart Foundation Health Promotion Research Group, Department of Public Health, University of Oxford, Oxford, UK; 2 SDU, University of California San Diego, La Jolla, USA; 3 Centre for Physical Activity and Nutrition, Auckland University of Technology, Auckland, New Zealand; 4 McCaughey VicHealth Centre for the Promotion of Mental Health and Community Wellbeing, the University of Melbourne, Parkville, Australia; 5 CLARITY: Centre for Sensor Web Technologies, Dublin City University, Dublin, Ireland

**Keywords:** SenseCam, Accelerometer episode, Context, Measurement, Physical activity, Sedentary behaviour

## Abstract

**Background:**

Accelerometers can identify certain physical activity behaviours, but not the context in which they take place. This study investigates the feasibility of wearable cameras to objectively categorise the behaviour type and context of participants’ accelerometer-identified episodes of activity.

**Methods:**

Adults were given an Actical hip-mounted accelerometer and a SenseCam wearable camera (worn via lanyard). The onboard clocks on both devices were time-synchronised. Participants engaged in free-living activities for 3 days. Actical data were cleaned and episodes of sedentary, lifestyle-light, lifestyle-moderate, and moderate-to-vigorous physical activity (MVPA) were identified. Actical episodes were categorised according to their social and environmental context and Physical Activity (PA) compendium category as identified from time-matched SenseCam images.

**Results:**

There were 212 days considered from 49 participants from whom SenseCam images and associated Actical data were captured. Using SenseCam images, behaviour type and context attributes were annotated for 386 (out of 3017) randomly selected episodes (such as walking/transportation, social/not-social, domestic/leisure). Across the episodes, 12 categories that aligned with the PA Compendium were identified, and 114 subcategory types were identified. Nineteen percent of episodes could not have their behaviour type and context categorized; 59% were outdoors versus 39% indoors; 33% of episodes were recorded as leisure time activities, with 33% transport, 18% domestic, and 15% occupational. 33% of the randomly selected episodes contained direct social interaction and 22% were in social situations where the participant wasn’t involved in direct engagement.

**Conclusion:**

Wearable camera images offer an objective method to capture a spectrum of activity behaviour types and context across 81% of accelerometer-identified episodes of activity. Wearable cameras represent the best objective method currently available to categorise the social and environmental context of accelerometer-defined episodes of activity in free-living conditions.

## Background

Insufficient levels of physical activity are associated with increased morbidity and mortality for a number of non-communicable diseases [1-3]. Understanding the determinants and barriers to physical activity behaviours, and the social and environmental context in which they occur, is important in designing interventions to positively change these behaviours [4,5]. Accurate measurement of the behaviour type and context of physical activity episodes is therefore important [6]. Examples of important context attributes of an episode of physical activity include: whether it occurs indoors or outdoors; the time of day it occurs; if it is alone or in companionship; and its domain (home, occupational, etc.) [7]. Currently, some of these attributes are subjectively measured via self-report which is prone to error associated with recall, comprehension, and social desirability bias [8,9].

**Figure 1:**
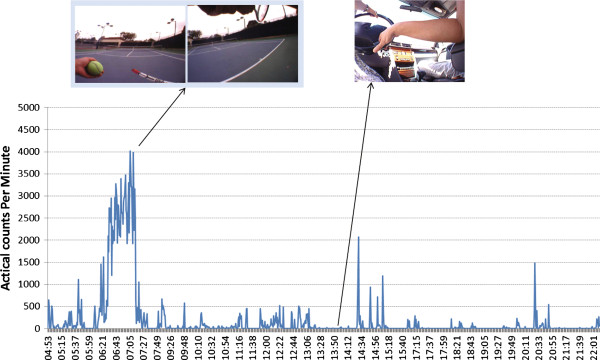
**SenseCam aligned with accelerometer trace.** Trace of Actical accelerometer counts from a single participant and day. Data from wearable cameras such as the SenseCam now allow an objective measurement of the type and context of physical activity behaviours the participant is engaged in.

It is challenging to objectively categorise the context of episodes of activity. Direct observation techniques are accurate, but are expensive [10] and can cause participants to change their typical behaviour. Accelerometers have been shown to be a valid and reliable objective measure of physical activity intensity [11,12]. Advancements in signal processing algorithms allow for the identification of some locomotive activity types [13]. However, hip worn accelerometers underestimate many types of activities that do not include central body movements. In addition accelerometers do not provide information on the context in which activities occur. Consider Figure 1, which illustrates a trace of a person’s activity counts over a day. Using only acceleration data it is impossible to identify signals that distinguish whether an activity occurs: indoors vs. outdoors; alone vs. in companionship, or at home vs. work. Global positioning system (GPS) devices can provide an objective measure of locational context for accelerometer-identified episodes of physical activity [14-16]. However GPS devices also have limitations: they suffer from signal loss in some indoor or underground locations, and around tall buildings [17]; use derived algorithms to estimate whether an activity occurs indoors/outdoors; and are unable to record whether the participant is alone or socially engaged. Ecological Momentary Assessment through cell phones is another method that researchers have used to attempt to capture more detail about activity and location attributes [18]. Yet these also rely on participants responding to a prompt and are reliant on accurate data entry by the participant. The challenge remains to objectively provide indoor/outdoor, social engagement, and domain context information for episodes of activity identified by accelerometers. To design successful interventions, accurate measurement of existing behaviour on what people are doing and when, as well as under what conditions, is critical [5]. This helps understand when and what types of interventions might be most successful in changing behaviour [4].

In this study we investigate the feasibility of a new technology to contextualise accelerometer data, namely the SenseCam [19]. The SenseCam is a wearable camera that automatically takes photos from a first person point of view[19]. The feasibility of using this device has been established for physical activity in the active travel domain [8]. Now we explore whether SenseCam can provide context information for episodes of accelerometer-assessed physical activity data, as illustrated in Figure 1. We have two primary research questions: 

1. Can wearable camera images be captured during accelerometer identified episodes of free-living activity?

2. Can accelerometer identified episodes be classified according to type and context attributes using wearable camera images?

The results of this study will be of benefit to researchers who use accelerometers to infer participant behaviour. It will allow for collection of more detailed information on the type and context of free-living behaviours participants are engaged in.

## Methods

### Recruitment of participants

A convenience sample of 52 participants was recruited in two countries: 15 from New Zealand (June 2011, winter) and 37 from the USA (June 2011, summer). In New Zealand 15 university workers were administered the devices on a Monday evening, and then wore them for 3 days from Tuesday morning to the end of Thursday evening. In the USA the same 3 day protocol was administered to 18 university workers, but this time capturing Friday-Sunday data. The following week another set of 19 university workers in the USA followed the same 3 day protocol, this time capturing Wednesday-Friday data. Although many studies use a 7 day monitoring protocol for physical activity, we were not trying to estimate typical weekly behaviour, but to merely capture sufficient examples of different types of activity behaviours.

### Ethical approval

Ethical approval for this study was granted by the ethics board in each of the three respective participating universities (AUTEC 11/114, May 25th 2011), (Ref No.: 111160/ UCSD August 4th 2011), (SSD/CUREC1A/10-054 Oxford, July 16th 2010). All participants signed an informed consent statement that was approved by the three aforementioned ethical committees. Participants in San Diego were financially compensated.

### Data collection

The SenseCam is a lightweight wearable camera worn via a laynard around the neck. It has a number of onboard sensors including: tri-axial accelerometer, magnetometer (much like a digital compass), ambient temperature, light level, and passive infrared (much like a home streetlight detecting presence of bodyheat). Images are captured based on a change in the aforementioned sensor values, resulting in images being taken approximately once every 20 seconds [20]. While the SenseCam has an onboard accelerometer, it is not hip- or wrist- mounted as is standard in the physical activity research community. Also the SenseCam accelerometer has not been validated for physical activity measurement.

The Actical (Mini-Mitter, Respironics Inc Company, Bend, OR) accelerometer was used to capture physical activity. These units contain a piezoelectric transducer that is sensitive to motion to 0.05g, and a microprocessor to convert accelerations to a unit termed activity counts. Evidence of the validity of physical activity intensity measurement in adults using these units has been established using indirect calorimetry [21]. Units were set to collect data at 15 second epochs (the shortest available epoch with these monitors).

All wearable devices were initialised on the same laptop, which was time synchronised with a world atomic clock^a^. This helped ensure that data collected across devices were correctly aligned.

Participants were supplied with an Actical accelerometer and a SenseCam wearable camera for 3 full days. They were asked to wear the devices for all waking hours each day and to go about their everyday free-living activities. In the USA, participants were provided with clothing adherence tape to secure the devices in place during intense exercise bouts. Participants were informed that they should remove the devices in settings where they felt it was not permitted or appropriate to be taking photographs. They also noted times when they wanted images removed and were given the opportunity to review their images and delete any they did not wish the researchers to see.

### Data processing

Using the manufacturer supplied software, the Actical 15 second epoch accelerometer data were downloaded and saved as a CSV file. Thereafter it was imported into a Microsoft SQL Server database [22]. A C#.NET computer program was written to select episodes of activity. Firstly, the data were aggregated into one-minute epoch values which is standard for an adult population [23,24]. Secondly, any instances with ten or more constant non-zero values were treated as spurious data to be removed [25]. Thirdly, episodes of non-wear time were identified where there were 60 consecutive minutes of zero counts per minute, with 2 minutes of grace between 0-100 counts allowed [24,26]. No wear-time criteria were applied, as the unit of analysis in this article is physical activity episodes, rather than an estimate of participants’ weekly levels of physical activity. This means that eligible episodes outside the 3 days of wear-time requested of participants could also be included. We used cut points to identify episodes of activity. To obtain a range of behaviour types and contexts we selected four intensities of activity: sedentary, lifestyle-light, lifestyle-moderate, and moderate-to-vigorous physical activity (MVPA). Each activity episode was at least 10 minutes in duration between threshold counts per minute values, with 2 minutes of grace allowed outside the defined threshold. This approach follows other episode selection algorithms [12]. As there is no published Actical lifestyle-light/moderate cut point similar to Matthews’ 760 for Actigraph [27], we interpolated a guide value of 565 counts per minute. The Actical device cut points we used were: sedentary (0-100) [24], lifestyle-light (101-564), lifestyle-moderate (565-1534), MVPA (1535+) [28]. Finally a random selection of up to 100 examples per each intensity level were selected for analysis.

**Figure 2:**
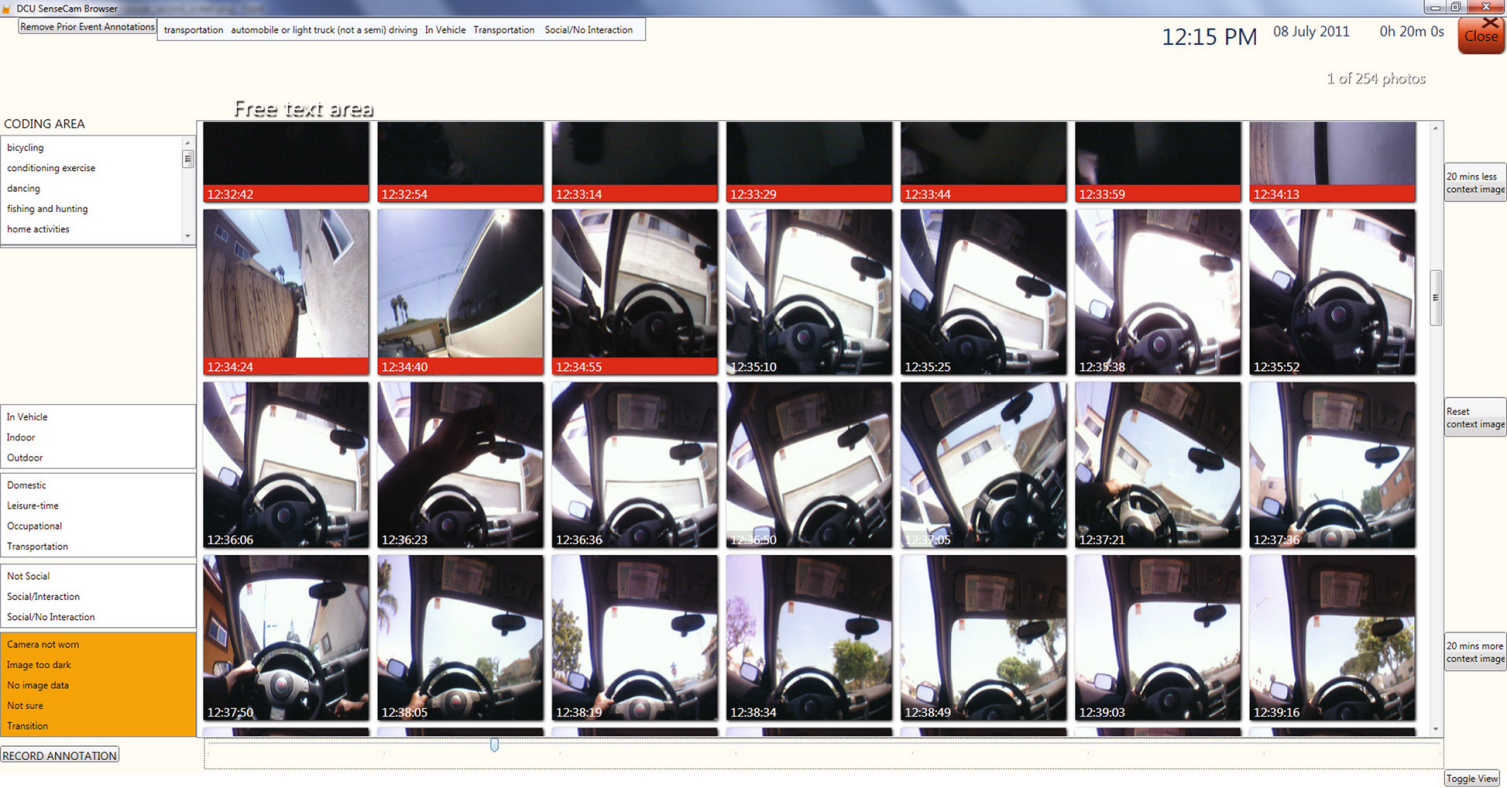
**Episode annotation using SenseCam images.** Overview of software developed to annotate SenseCam images. The left hand side displays the initial screen which lists all the episodes identified from Actical data. Once the researcher has clicked on an individual episode, a new screen opens up which is displayed on the right hand side of the figure. This screen allows the researcher to view all the images associated with the given episode, and then manually categorise its behavioural type and context. This particular example shows a participant driving a vehicle during the selected episode. The red-bordered images represent photos taken before the selected episode.

The SenseCam wearable camera data were downloaded from the device into a custom software application, which can be freely downloaded^b^[29]. The software was modified to only show accelerometer identified episodes of activity and the images associated with them (Figure 2). Once an episode was selected in the browser, a new screen appeared showing all the SenseCam images recorded between the start and end time of the episode. To support understanding of the episode, the software also presents images occurring 15 minutes before and after the given episode. The researcher had the option of increasing or decreasing the number of images to either side of episodes, to help better understand what activities occurred before or after. These surrounding context images were highlighted with a red border so the researcher knew not to consider them when annotating the episode. The software allows for the annotation of each episode (see Figure 2).

### Annotation protocol

Episodes were annotated on six dimensions. The first two dimensions were the physical activity behaviour type and subtype, using the 21 categories and 821 subcategories suggested by the 2011 Compendium of Physical Activities [30]. Following some iterations of training a classification technique was agreed upon. Given the large number of potential subcategory annotations available, a general guideline given to annotators was that the most descriptive subcategory should be selected, but if in doubt the relevant Taylor code [31] should be used. The third dimension was whether the bout had predominantly occurred indoors or outdoors. The fourth dimension recorded the domain in which the activity occurred, using the following CDC suggested categories: occupational, domestic, transportation, leisure-time^c^. The fifth dimension recorded if the participant carried out their episode of activity alone, in a social environment with no interaction (e.g., at shopping mall), or in direct social engagement (e.g., playing tennis with a friend). The final dimension was an optional comment field to describe why an episode could not be confidently annotated. Episode images, in addition to the surrounding contextual images before and after the bout, provided visual clues as to the activity type. Visual clues included: putting on trainers before a run which contains blurred images; putting on a backpack before a hike; multiple pictures of the same human or animal throughout the episode; pictures of an additional consistent shadow of another companion, etc. Prosser and Loxley recognise that *“the usefulness of visual evidence depends on the [researcher’s] skill in drawing data from the images that can form the basis of interpretations about what is happening”*[32]. Therefore two researchers, who underwent a one hour training course in ethics in the University of Oxford, independently annotated a random subsample of 25% of episodes. An inter-rater reliability analysis using the Kappa statistic was performed to determine consistency among raters for classification across the aforementioned dimensions of: activity, activity subcategory, inside/outside, domain, alone/accompanied, and comment (optional).

### Data analysis

The annotation information was stored in the SenseCam software’s Microsoft SQL Server supporting database. The annotated episodes were transferred for further analysis to PASW Statistics 19.0 (SPSS Inc., Chicago, IL, USA). The variables recorded for each episode were: participant ID and location (from participant informatiom sheet), activity type, activity subcategory (from Physical Activity Compendium and SenseCam), indoor/outdoor, domain, social interaction status (from SenseCam), start/end times, duration (from Actical), MET value for activity (from Physical Activity Compendium), and average accelerometer intensity in counts per minute (from Actical). Summary statistics were then generated by activity type to investigate how many accelerometer identified episodes could be classified. Thereafter further summary statistics were generated grouping by activity type to investigate the context (in terms of indoor/outdoor, domain, and social interaction status) surrounding each activity type.

## Results

Of the 52 participants, 3 from New Zealand were removed as the firmware on the device was accidentally reset by these participants. Thus 49 participants were included for data analysis. No spurious Actical data were found, but 393 episodes of non-wear time were identified and removed prior to analysis. Across the 212 days of valid Actical data from 49 participants, there were 3017 episodes identified using the accelerometer processing techniques. Table 1 profiles the 386 randomly selected Actical episodes that were annotated for further analysis. Total annotation time across all 386 episodes was approximately 6 hours 45 minutes, an average of approximately 63 seconds (CI: 41 - 86 sec) to annotate each 10 minute episode. A subset of 93 episodes (22 MVPA, 28 lifestyle-moderate, 18 lifestyle-light, 25 sedentary) from 13 participants were annotated independently by two researchers. The inter-rater reliability was 0.962 for activity type, 0.492 for activity subtype, 1.000 for indoor/outdoor, 0.903 for domain, and 0.621 for social interaction status.

**Table 1:** Actical accelerometer episodes selected for annotation using SenseCam images

**Episode**	**Num**	**Mean**	**(95% CI on**
**Type**	**episodes**	**duration**	**episode duration)**
MVPA	86	17.0 min	(14.7 - 19.3 min)
Lifestyle-moderate	100	15.4 min	(13.6 - 17.2 min)
Lifestyle-light	100	14.9 min	(10.1 - 19.8 min)
Sedentary	100	24.0 min	(20.6 - 27.5 min)

### 1. Can wearable camera images be captured during accelerometer identified episodes of activity?

From the 49 participants with valid data, 441,143 SenseCam wearable camera images were captured. From the 386 randomly selected episodes, 19% (n=75 episodes, 16 MVPA, 16 lifestyle-moderate, 16 lifestyle-light, 27 sedentary) could not have their behaviour type and context annotated. Reasons for this are outlined in Table 2. Across the 81% of 10 minute episodes (n=311: 70x MVPA, 84x lifestyle-moderate, 84x lifestyle-light, 73x sedentary) that did have identifiable activity images, 20,587 images were captured, with one image being captured on average every 17 seconds (95% CI:15-20 sec).

**Table 2:** A failure analysis on the 75 Actical episodes that could not be annotated using SenseCam

**Reason**	**MVPA**	**Lifestyle-**	**Lifestyle-**	**Sedentary**
		**moderate**	**light**	
No associated	10	15	13	19
images				
Images too	4		1	5
obscured				
Images too dark	1	1	2	3
Camera not worn	1			

From the randomly selected data, we compared the 75 (16 MVPA, 16 lifestyle-moderate, 16 lifestyle-light, 27 sedentary) episodes that could not be annotated with the 311 that could be annotated. Table 3 displays the differences by each Actical intensity level. The only noteworthy difference was that the sedentary episodes that could not be annotated had a slightly lower accelerometer count score [3.1 cpm (95% CI: 1.7 - 4.6 cpm)] than those that could be annotated [7.0 cpm (95% CI: 5.2 - 8.9 cpm)]. Of the 10 participants who did not wear the SenseCam for some MVPA episodes, 6 of them did wear the SenseCam for other recorded MVPA random selection episodes. The results for other episodes that could not be annotated from the random selection for other categories are similar: 9 participants had lifestyle-moderate episodes that could not be annotated, but 6 of those did wear the SenseCam for other recorded lifestyle-moderate randomly selected episodes; 6 from 8 participants had a similar story with lifestyle-light episodes; and 11 from 18 participants with Sedentary episodes.

**Table 3:** A comparison of the Actical accelerometer identified episodes that could be annotated using the SenseCam (n=311) vs. those that could not (n=75)

	**Duration**		**Intensity**	
	**(mins ± SD)**		**(cpm ± SD)**	
**Episode Type**	**Not-Annotatable**	**Annotatable**	**Not-Annotatable**	**Annotatable**
Sedentary	27.1±22.4	22.9±15	3.1±3.7	7±8.1
	(n=27)	(n=73)	(n=27)	(n=73)
Lifestyle-light	11.9±4.2	15.5±26.5	277.1±51.4	275.3±64.4
	(n=16)	(n=84)	(n=16)	(n=84)
Lifestyle-moderate	14.4±6.4	15.6±9.5	978.4±151.1	967.5±147.1
	(n=16)	(n=84)	(n=16)	(n=84)
MVPA	18.6±10	16.6±10.8	3876±2243.4	3127.7±1680.5
	(n=16)	(n=70)	(n=16)	(n=70)
TOTAL	19.4±15.8	17.5±17.3	1096±1811	1041±1425
	(n=75)	(n=311)	(n=75)	(n=311)

### 2. Can accelerometer identified episodes be classified according to their context attributes using wearable camera images?

The 311 Actical identified episodes were categorised into 12 Physical Activity Compendium categories and 114 subcategories as detailed in Table 4: 30% of bouts were bicycling, 23% walking, 14% occupation, 13% home activities, etc. The context surrounding where episodes took place is detailed in Table 5. 59% of episodes were outdoors, 39% indoors, and 3% were in vehicle. With respect to the domain where the episodes occurred, 33% were leisure time, 33% transportation, 18% domestic, and 15% occupational as detailed in Table 5. 45% of episodes were in non-social situations, 33% involved direct social interaction, and 22% were in social situations where the participant wasn’t necessarily involved in direct engagement.

**Table 4:** Actical accelerometer episodes (column headings) annotated into PA Compendium categories using SenseCam wearable camera images

	**Sedentary**	**Lifestyle**	**Lifestyle**	**MVPA**	**Duration**
		**Light**	**Moderate**		
	**Min ± SD**	**Min ± SD**	**Min ± SD**	**Min ± SD**	**Min ± SD**
Bicycling	−	14.8±8.1	15.5±9.1	14.7±4.2	15.2±8.5
(5x subcategories)		(*n*=28)	(*n*=59)	(*n*=6)	(*n*=93)
Walking	−	10.3±1.5	11.1±2.2	16.2±11	15±9.9
(9x subcategories)		(*n*=3)	(*n*=14)	(*n*=56)	(*n*=73)
Occupation	27.3±17.3	10±1	16±0	−	25.5±17
(2x subcategories)	(*n*=37)	(*n*=3)	(*n*=2)		(*n*=42)
home activities	11±2.2	18.4±40.4	14±7.1	−	17.5±37.4
(19x subcategories)	(*n*=4)	(*n*=35)	(*n*=2)		(*n*=41)
Miscellaneous	20.4±8.8	10.8±3.6	9±.	−	17.3±8.8
(5x subcategories)	(*n*=15)	(*n*=6)	(*n*=1)		(*n*=22)
self care	24.4±16.5	24±.	−	−	24.3±15.4
(2x subcategories)	(*n*=8)	(*n*=1)			(*n*=9)
sports	−	−	32±14.8	29.8±12.1	31±12.9
(3x subcategories)			(*n*=5)	(*n*=4)	(*n*=9)
transportation	15±3.9	12.3±3.5	−	−	14.1±3.8
(2x subcategories)	(*n*=6)	(*n*=3)			(*n*=9)
conditioning exercise	−	15±.	9±.	11.5±2.9	11.7±2.9
(2x subcategories)		(*n*=1)	(*n*=1)	(*n*=4)	(*n*=6)
home repair	9±0	13±.	−	−	10.3±2.3
(3x subcategories)	(*n*=2)	(*n*=1)			(*n*=3)
lawn & garden	−	−	−	9.7±0.6	9.7±0.6
(3x subcategories)				(*n*=3)	(*n*=3)
Inactivity	11±.	−	−	−	11±.
(1x subcategory)	(*n*=1)				(*n*=1)

**Table 5:** Actical accelerometer episodes (column headings) annotated into context categories using SenseCam wearable camera images

	**Sedentary**	**Lifestyle**	**Lifestyle**	**MVPA**	**Duration**
		**Light**	**Moderate**		
	**Min ± SD**	**Min ± SD**	**Min ± SD**	**Min ± SD**	**Min ± SD**
In Vehicle	14.8±4.3	12.3±3.5	−	−	13.9±4
	(*n*=5)	(*n*=3)			(*n*=8)
Indoor	24±15.5	18±37.4	19.4±12.5	11.5±2.9	21.2±24.9
	(*n*=65)	(*n*=41)	(*n*=10)	(*n*=4)	(*n*=120)
Outdoor	12±2.6	13.2±7	15.1±9	16.9±11	15.3±9.4
	(*n*=3)	(*n*=40)	(*n*=74)	(*n*=66)	(*n*=183)
Domestic	20.3±11	17.6±38.3	19±	9±.	18.2±32.1
	(*n*=16)	(*n*=39)	(*n*=1)	(*n*=1)	(*n*=57)
Leisure-time	17.4±8.1	13.5±6.4	18.2±12.4	19±12.5	17.4±10.9
	(*n*=14)	(*n*=23)	(*n*=22)	(*n*=45)	(*n*=104)
Occupational	26.7±17.8	9.7±1.2	13.7±4	11.5±3.5	24.2±17.3
	(*n*=39)	(*n*=3)	(*n*=3)	(*n*=2)	(*n*=47)
Transportation	15.5±4.7	14.5±8	14.6±8.4	12.4±4.4	14.2±7.5
	(*n*=4)	(*n*=19)	(*n*=58)	(*n*=22)	(*n*=103)
Not Social	24.3±16.9	12.2±3.3	14.2±8.7	18.8±14.6	16.8±12.2
	(*n*=33)	(*n*=41)	(*n*=42)	(*n*=24)	(*n*=140)
Social/Interaction	20.4±11.6	19.7±43.8	18.7±12.8	17.7±9.4	19.2±25
	(*n*=28)	(*n*=30)	(*n*=18)	(*n*=28)	(*n*=104)
Social/No Interaction	24.9±17	16.3±10.3	15.6±7.7	12±3.2	16.4±10.5
	(*n*=12)	(*n*=13)	(*n*=24)	(*n*=18)	(*n*=67)

## Discussion

In this study we sought to investigate whether wearable cameras could complement existing accelerometry measures to objectively identify behavioural type and context information across a range of activity episodes. We found that 81% (311 out of 386) of the randomly selected episodes that were identified by Actical could be identified using the SenseCam. Furthermore of those identified episodes, it was possible to objectively determine the type of behaviour the participant was engaged in (e.g., 30% of all episodes were categorised as walking). For each type of behaviour (e.g., walking), it was also possible to determine the context in which it occurred. For example, in our selection of participants, all walking took place outdoors (n=73), with 53% (n=39) of it in the leisure-time domain. 59% (n=23) of leisure-time walks contained social interaction, with the most common (48%, n=11) subactivity behaviour type being Physical Activity Compendium category 17012, *“backpacking, hiking or organized walking with a daypack”*.

Such detailed annotation is possible through examining not only the images of the episode in question, but also the images preceding and succeeding the 10 minute episodes. This allows us for example to determine whether a walk finishing at workplace vs. at local park should have its domain annotated as transportation vs. for leisure. Although the annotation of episodes is manual in this article, these annotations will help form a basis to train future machine vision automated techniques [33]. This is the first study using digital image capture to complement accelerometer based behaviour assessment. We show that the technique is feasible and enhances the data that can be collected in a free-living situation.

### Limitations

Twenty-one percent (n=75 episodes, 1453 min) of episodes identified by the Actical accelerometer could not be classified (see Table 2). This was mostly due to there being no associated images (76% of un-annotated episodes), which indicates that compliance of wearing the device can be improved. Out initial hypothesis was that this is due to the SenseCam swinging uncomfortably while engaged in MVPA activities. However these 57 episodes across 23 of the participants when the units were removed, were from a range of intensity levels: 19 sedentary, 15 lifestyle moderate, 13 lifestyle light, and 10 MVPA. Further work is needed to investigate the type of activities where participants do not feel that a wearable camera is either comfortable or practical to wear.

This work relies on manual annotation of the data, but this is a necessary first step towards developing automated computer vision techniques in future [33]. Currently it takes approximately 63s (CI: 41s - 86s) to annotate each MVPA bout. However this work is focused on the feasibility of identifying the behavioural type and context information. Building a system to automatically recognise the characteristics of physical activity behaviours [34] based on the manual annotations from this work will reduce future researcher burden and time spent on data analysis.

Using the guidelines of Landis & Koch, the inter-rater reliability for manually annotating Physical Activity Compendium category (0.962), indoor/outdoor (1.0), and domain (0.903) could be considered as almost perfect agreement [35]. The social interaction status (0.621) could be considered as substantial inter-rater agreement, while the Physical Activity Compendium subcategory annotation (0.492) could be considered as moderate. On closer inspection of the subcategory annotations, two categories were confused most often: four instances of 17160:*“walking for pleasure (Taylor code 010)”* vs. 17165: *“walking the dog”*; and seven instances of 17012:*“backpacking, hiking or organized walking with a daypack”* vs. 17080:*“hiking, cross country (Taylor Code 040)”*. If specific guidelines were given on classifying between categories 17160/17165 and 17012/17080, subcategory inter-rater agreement would then have been 0.811 (hence considered as almost perfect). While the focus on this article has been on introducing the SenseCam as a novel method of identifying the behavioural type and context of activities, future efforts should focus on publishing a detailed annotation guidebook. For example annotating between walking and running activities could also present issues, as from the participant’s perspective both appear quite similar visually. A possible means to distinguish between these two activities would be to reference the accelerometer intensity data. Other future efforts could focus on introducing more sensitive context categories.

**Figure 3:**
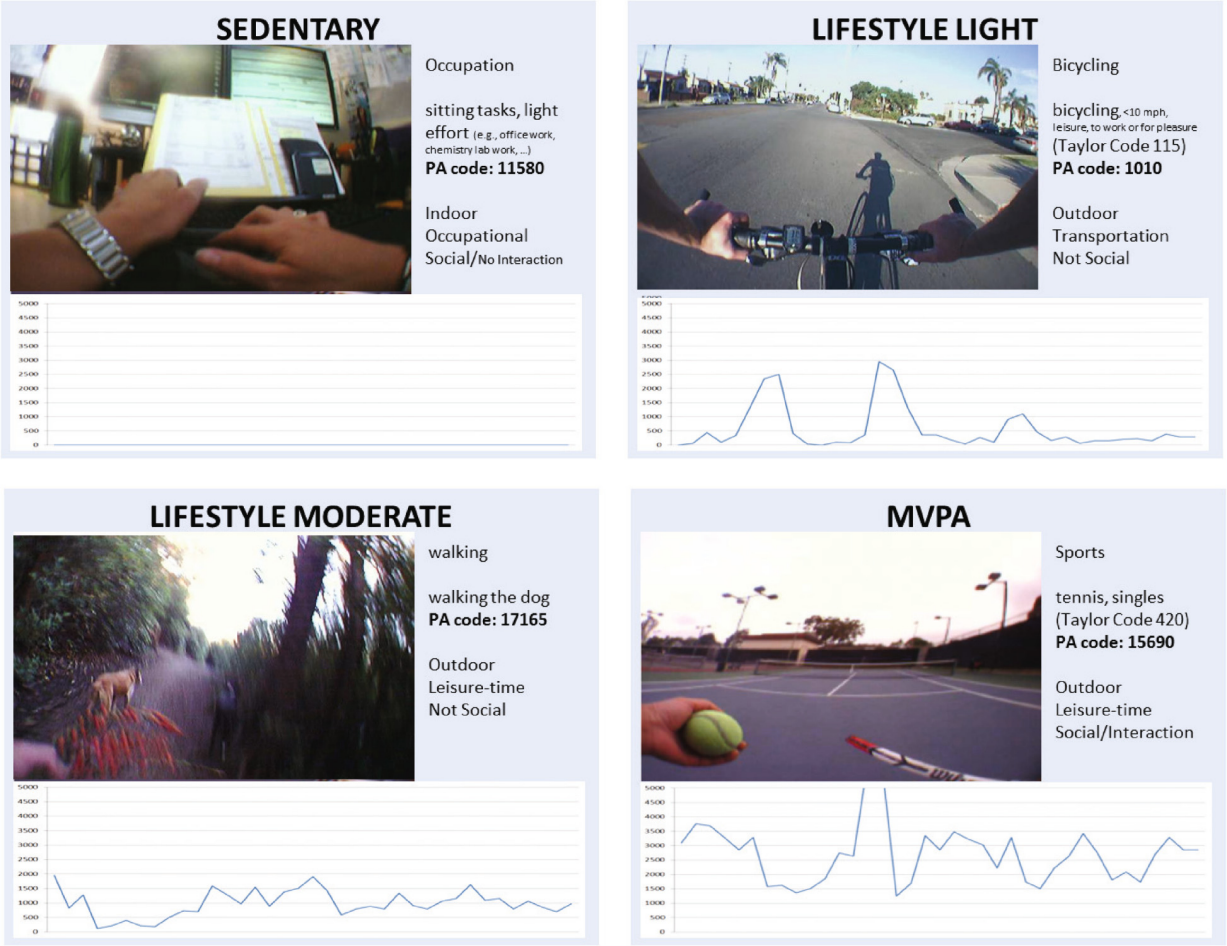
**Sample of annotated episodes using SenseCam.** A selection of episodes of activity that were categorised using SenseCam images. The Physical Compendium category, contextual annotation, and associated Actical counts per minute is displayed with each example episode.

We believe accelerometers are most appropriate for identifying the start and end times of episodes of behaviour. This is due to automated image analysis being difficult due to the semantic gap, where digital representations of image pixels do not easily transfer to semantic descriptions of what an image is about [29,36]. The strength of wearable camera images is in categorising type and context information across a range of episodes (see Figure 3). The method we have introduced in this paper is agnostic of choice of accelerometer episode identification algorithm. We used cut points to identify episodes of activity, but signal processing techniques could also have been used to identify these episodes [37].

### Future work

The annotations of episodes in this paper should be used as training data for machine vision and accelerometer classifiers. Such classifiers have been applied to SenseCam wearable camera data [34], but were on a small constrained dataset. Future work should evaluate how accurately the automated techniques can replicate our manual annotations.

Wearable cameras allow for detailed behaviour type and context analysis across a range of categories. For example we identified eight Physical Activity Compendium categories as outdoor episodes, and ten categories as indoor episodes. The outdoor categories were: 90 bicycling, 73 walking, 6 home activities, 5 sports, etc. The indoor categories were: 42 occupation, 35 home activities, 8 self care, 6 conditioning exercise, etc. An analysis combining such objectively identified behavioural type and context information from free-living scenarios may lead to a better understanding of determinants driving physical activity behaviours.

As this article has been focused on classifying the type and context of accelerometer identified episodes, there is an implicit assumption the accelerometer captures all episodes of interest. There is also a reliance on the accuracy of its episode identification algorithm for sedentary vs. lifestyle-light vs. MVPA etc. Future work should investigate such intensity classification techniques from accelerometer data using sensitivity and specificity analysis. Another issue not covered in this article is that when synchronising the onboard firmware clocks of the SenseCam and Actical devices, checks should be identified that there is no *“drift”* between the onboard clocks on these devices. Future investigations should investigate if there is a time discrepancy between both onboard device clocks after prolonged durations (e.g. 24 hours, 7 days, etc.).

Wearable camera images may be more appropriate to determine the range of types of sedentary behaviour. Accelerometers can identify episodes of sedentary behaviour, but are not suitable in determining the specific type of non-locomotive activity participants are engaged in [38]. Manually annotating wearable camera images in this study, it was possible to further break down sedentary episodes into 7 Physical Activity Compendium categories and 16 subcategories (Table 4): 37 occupation, 8 self care, 6 transport, 4 home activities, 2 home repair, 1 inactivity, 15 miscellaneous. Using this method of combining type and context information on populations of interest could lead to better understanding the determinants driving sedentary behaviours.

More detailed analysis of activity behaviour types should be conducted in future. This study identified 12 categories from a possible list of 21 as covered by the 2011 compendium of physical activities. In addition 114 Physical Activity Compendium subcategory behaviours were identified. Larger scale studies in future may identify and test a greater diversity of activity behaviours.

## Conclusions

The type and context of behaviour episodes can be identified through manual annotation of wearable camera images. This verifies that machine vision classifiers should be evaluated on whether they can automatically replicate these manual annotations. If this is satisfied future studies using accelerometers could consider the use of wearable cameras to objectively categorise and contextualise accelerometer identified episodes of activity, regardless of choice of episode or bout type identification algorithm. The wearable camera should not be viewed as a replacement device for the hip-mounted accelerometer, but instead as a complementary source of information to provide much needed contextual information. This will enable researchers to better understand human behaviour using an objective free-living behaviour, thus providing better quality information to devise appropriate public health interventions [4]. Future studies using both of these devices together will likely provide better objective measurement of episodes of interest in terms of: type, context; intensity (from accelerometer, and also cross-referencing Physical Activity Compendium); time, and duration (from either device).

## Endnotes

^a^http://www.worldtimeserver.com/atomic-clock/^b^http://sensecambrowser.codeplex.com^c^http://www.cdc.gov/physicalactivity/professionals/data/explanation.html

## Abbreviations

MVPA: Moderate to Vigorous Physical Activity.

## Competing interests

The authors declare that they have no competing interests.

## Authors’ contributions

Authors PK, AD, MO, JK, and HB carried out the data collection. Authors AD and AH annotated all the accelerometer episodes. Author AD developed the software tools and processes to clean and align SenseCam and accelerometer data sets. Author AD led the writing of the article, with numerous contributions from authors CF, PK, MO, AH, JK, SM, and HB. All authors read and approved the final manuscript.
